# The Influence of the Sous Vide Cooking Time on Selected Characteristics of Pork Lion

**DOI:** 10.3390/molecules28166102

**Published:** 2023-08-17

**Authors:** Ryszard Rezler, Mirosława Krzywdzińska-Bartkowiak, Michał Piątek

**Affiliations:** 1 Department of Physics and Biophysics, Faculty of Food Science and Nutrition, Poznań University of Life Sciences, Wojska Polskiego 38/42, 60-624 Poznań, Poland; 2 Department of Meat Technology, Faculty of Food Science and Nutrition, Poznań University of Life Sciences, Wojska Polskiego 31/33, 60-624 Poznań, Poland

**Keywords:** colour, meat, rheological properties, sous vide, texture analysis, thiamine

## Abstract

The aim of this study was to determine the effect of sous vide and pressure-cooker cooking of pork muscles (*Longissimus lumborum*) on the physicochemical and technological characteristics of pork. The study included an analysis of the basic composition, colour, texture, sensory evaluation, nutritional value (vitamin B1 content), and rheological properties of meat cooked at 60 °C for 6–18 h and, for comparison, in an autoclave at 121.1 °C. The heating conditions affected the weight loss, colour, thiamine content, texture, and rheological properties of the meat. As the heating time increased, the texture determinants of firmness and chewiness decreased, which resulted in softer meat. The differences in the rheological properties of the sous-vide- and autoclave-cooked meat resulted from the different organisation of the spatial matrix of proteins and changes in the structure of muscle fibres caused by the high temperature.

## 1. Introduction

One of the most popular of methods used in the production of ready-to-eat food is sous vide [1,2]. The sous vide (SV) cooking process comprises the vacuum packing of raw food ingredients (vegetables, fruits, and especially meat) into thermostable vacuum bags or containers and heating them for a long time at a specific temperature [3,4].

Research on the effect of SV on the quality of cooked meat at a wide range of temperatures (50–100 °C) for various periods of time (3–24 h) [5,6] has shown that, thanks to the SV method, the natural taste of meat can be preserved, and the loss of its nutritional value can be limited. The SV method is also characterised by a greater retention of the minerals, metals, and vitamins contained in meat. Unlike traditional cooking methods (cooking under atmospheric pressure), the SV method positively affects the quality and nutritional properties of meat [7]. In addition, cooking at low temperatures (up to 65–70 °C) causes fewer colour changes in raw meat [8]. Due to the limited loss of water in the SV method the food remains juicy, and ready-made meals have good sensory properties [9]. Temperature and time are very important parameters with considerable influence on the quality of SV-cooked meat [10]. Chefs commonly recommend cooking beef, pork, and lamb at around 58–63 °C for 4–18 h [4,11]. Low temperature and long cooking time increase the tenderness of different kinds of meat, such as beef [12,13], pork [14], and lamb [15]. Among various types of meat, pork loin plays a special role in Polish cuisine. Fried and roasted pork loin dishes are considered traditional. Ready-to-eat meat prepared in this way is commercially available. However, the heat treatment of meat causes physicochemical changes in its components, such as protein denaturation, contraction of muscle fibres, collagen solubilisation and gelation, and significant water loss [16]. These changes affect the meat texture. While the solubilisation and gelation of collagen has a softening effect, most of the changes occurring during cooking in proteins, mainly myofibrils, increase meat toughness [17]. Due to the loss of moisture, the meat is dry and loses various nutrients, including vitamins and minerals [18]. As a result, there is a low demand for such products. The SV method is an alternative procedure, thanks to which the quality and commercial attractiveness of thermally treated pork can be maintained or even improved [19].

As there is little information on how the combination of low temperature and long heating time affects the properties of sous-vide-cooked pork, extensive research on the physicochemical properties, texture, and content of nutrients (vitamin B1) in pork was undertaken. In order to determine the optimal SV state (temperature–time), tests were conducted within a temperature range of 50–85 °C over a wide range of heating times (4–18 h).

Both the instrumental texture analysis and sensory analysis showed that, in comparison with the other variants, the pork (*Longissimus lumborum* muscle) cooked with the SV method at 60 °C had the most attractive and acceptable features for consumers. It was noticeably juicier and more tender. Therefore, our study discusses the results of the experiment conducted on this variant with a wide range of heating times (6–18 h). At the same time, the authors attempted to explain the mechanisms determining the observed rheological and textural properties of the SV-cooked meat, because the vast majority of publications on meat cooked with this method either omit this issue or discuss it very superficially.

## 2. Results and Discussion

### 2.1. Basic Composition

The basic composition (protein, fat, water, and solids-non-fat content) of the SV-cooked meat samples is shown in Table 1.

The raw meat had the highest water content. Its values were significantly different from the other variants of cooked meat. On the other hand, the meat cooked for 30 min at 121.1 °C in an autoclave had the lowest water content. It was statistically significantly different from the samples heated with the SV method and the raw meat. The SV heating decreased the water content in the samples when heated for up to 8 h. At further heating stages, i.e., 8 h, 10 h, 12 h, 14 h, 16 h, and 18 h, the water content remained at the same level. The lower water content in the samples heated in the autoclave resulted from a much greater contraction of muscle fibres caused by the high temperature of thermal treatment, which resulted in a greater loss of water from the muscles.

The analysis of the results showed that the meat sample cooked in the autoclave for 30 min had the highest protein content, which was statistically significantly different from the protein content in the other variants. The raw meat had the lowest protein content, which was statistically significantly different from the other variants. The protein content in the SV samples heated for 6 h, 8 h, 10 h, and 12 h ranged from 26.50% to 27.22%. There was a statistically significant increase in the protein content in the sample heated for 14 h. This value was similar in the samples heated for 16 h, whereas the protein content in the sample heated for 18 h was slightly lower.

Changes in the protein content of meat are also caused by the loss of water. Since the water loss in the samples heated in the autoclave was higher, they had higher protein content than the raw meat or meat cooked for 6 h (Table 1). The sample cooked for 6 h had the lowest fat content, which was significantly different from the fat content in the other variants. As the SV heating time increased, so did the fat content in the samples cooked for 6–10 h (statistically significant differences). Further heating up to 12 h resulted in a slight increase in the fat content, but the difference was not statistically significant. There was a statistically significant decrease in the fat content in the successive meat heating variants; it amounted to 3.31% in the sample cooked for 16 h. The total meat composition and fat content are the main determinants of meat quality. Differences in the fat content between the samples may have been caused by the heterogeneous structure of the muscle. As the heating time of the meat increased, the fat melted and decomposed, whereas the weight loss of the product increased. The raw meat had the lowest fat-free dry matter content, which was significantly different from the other variants. The meat cooked in the autoclave at 121.1 °C for 30 min had the highest fat-free dry matter content, which was significantly different from the SV-cooked and raw meat samples. This effect was caused by a greater loss of water and other meat components (myofibrillar and sarcoplasmic proteins, collagen, lipids, vitamins, minerals, and flavour compounds).

### 2.2. Cooking Loss

Table 1 shows the mass losses in the meat samples after SV cooking at 60 °C for 6–18 h, and in the meat samples cooked in the autoclave (121.1 °C, 0.2 MPa).

Within the whole range of SV cooking times, the weight loss in the meat samples ranged from 23.19% to 26.72% and tended to increase slightly along with the cooking time (*p* < 0.05). The highest weight loss of 33.87% was observed in the meat samples cooked in the autoclave.

The loss of meat mass during cooking is caused by the contraction of muscle fibres and connective tissue, as well as the direction of heat conduction [20,21]. At about 60 °C the direction of thermal shrinkage to the muscle fibre axis mostly changes from transverse to longitudinal [22]. Transverse contraction, which is attributed to the expansion of the space between the muscle fibres and the endomysium, has a minimal effect on the loss of water and other meat components. Most of the meat weight loss during cooking is caused by the longitudinal contraction of the muscles as a result of denaturation of connective tissue proteins, mainly myofibrillar proteins and collagen. Myofibrillar contraction reduces the muscle’s ability to retain water. The contraction of the connective tissue of the perimysium is caused by the compression of muscle fibre bundles, which leads to the expulsion of a larger volume of fluid from the muscle [16,23].

The duration and temperature of cooking clearly influence the capacity of the meat to retain water. Many researchers observed greater weight losses in meat as the heating temperature increased, regardless of the meat type and thermal processing method [19,24].

In our study, the weight loss in the SV-cooked meat samples (*Longissimus lumborum* pork muscle) tended to increase within the temperature range of 60–85 °C (the results have not been presented in this manuscript). The lowest weight loss was observed in the variant heated at 60 °C. This value differed significantly from the other variants in the experiment.

The amount of weight loss in the SV heat treatment is also influenced by the heating time.

Hwang et al. [14] analysed the effect of temperature (50 °C, 55 °C, and 60 °C) and time (12 h and 24 h) on the weight loss in SV-cooked pork loin. The study showed that the weight loss during cooking tended to increase along with temperature, but longer cooking times at 50 °C resulted in a smaller loss. When the cooking temperature was higher than 55 °C, the cooking time had no effect on weight loss in the meat samples. However, after cooking at 60 °C for 24 h the weight loss was usually higher than after 12 h. Jeong et al. [24] analysed the effect of SV cooking conditions (temperature and time) on the properties of pork ham and observed a similar dependence. The amount of meat weight loss was influenced by temperature to a greater extent than by the cooking time. The study also showed that when the cooking time was shortened to 2 h the weight loss was lower, despite the temperature increase to 100 °C.

In our study the weight losses in the SV meat samples cooked at 60 °C were equal and lower than for the other heating variants (60–85 °C). The results of our experiment are in line with the observations made by other researchers.

As mentioned above, most of the water in the muscles can be found in myofibrils and between myofibrils, as well as between myofibrils and the sarcolemma [22]. Heating the muscle causes denaturation of meat proteins, including myofibrillar and collagen proteins. It results in the contraction and subsequent solubilisation of collagen. These heat-induced structural changes in the muscles result in fluid loss during cooking [25]. The denaturation and shrinkage of meat protein largely depend on the heating temperature. The weight losses in the SV-cooked muscle samples were smaller than in the autoclave-cooked samples, mostly because of the lower temperature of the heat treatment (60 °C). In consequence, both protein coagulation and collagen solubilisation were limited [26].

A high temperature (121.1 °C) causes the structural disintegration of myofibrils. Their structure is considerably loosened during cooking due to progressive changes in the intramuscular connective tissue, caused by the much higher intensity of the denaturation of myofibrillar and collagen meat proteins. As a result, muscle contraction is much greater than at lower temperatures. Such changes in the muscle structure cause a much greater loss of fluids. This effect is manifested by the increase in the dry matter content, especially the share of proteins in the meat composition (Table 1) [25].

### 2.3. Colour Analysis

The change of the meat colour during cooking is an important effect, influencing the assessment of its quality during consumption. The colour of a piece of meat cooked at a certain temperature depends on how quickly it reaches that temperature and how long it is held at that temperature. The faster it reaches the temperature, the redder it is, and the longer it is held at a certain temperature, the lighter it becomes [15].

The colour changes that occur during cooking reflect heat-induced changes in the muscle pigment, myoglobin. Denaturation and aggregation of myofibrillar and sarcoplasmic proteins are also important factors affecting the assessment of the colour of cooked meat. Changes in these proteins significantly affect the amount of light scattered [27].

Our research showed that both the SV and autoclave heating of pork significantly influenced its colour. The raw meat had the lowest value of the L parameter, determining the brightness of the finished product, and its values were statistically significantly different from the other variants. The duration of the SV heating of pork at 60 °C did not significantly affect the colour parameters, i.e., L* lightness, a* redness, and b* yellowness. Only the samples cooked in the autoclave at 121.1 °C had higher a* and b* values, but these had a lower L* value (Table 2).

The raw meat had a negative value of the a* parameter. By contrast, the value of the a* parameter in all other time variants (6–18 h) ranged from 0.16 to 0.70. The values of this parameter did not differ significantly between the samples. On the other hand, the largest statistically significant difference from the other variants was observed in the meat cooked in the autoclave.

The meat cooked in the autoclave at 121.1 °C for 30 min had the largest share of the yellow colour, whereas the raw meat had the lowest. A similar trend was observed in values of C*. The SV meat samples cooked at 60 °C for 6 h, 8 h, 10 h, 12 h, 14 h, 16 h, and 18 h did not differ statistically significantly in the share of yellowness (Table 2).

Sanchez del Pulgar et al. [28] observed that the cooking of meat at moderate temperatures, even for a very long time, did not affect the proportion of the red colour. Our analysis showed that the SV samples cooked at 60 °C for 5 h and 12 h had higher values of the L* parameter than those cooked at 80 °C for the same time or cooked traditionally. Similarly, Roldán et al. [29] observed that lamb samples cooked at 60 °C for different time periods had slightly higher L* values than those cooked at higher temperatures (70 °C or 80 °C).

Jeong et al. [24] used the SV method and different percentages of vacuum (98.81% or 96.58%) to heat pork ham at 61 °C and 71 °C for 45 and 90 min. The researchers observed higher values of the L* parameter as the temperature and heating time increased. This may have been caused by the fact that the higher water content in the cooked meat samples allowed light to penetrate deeper into the tissue and resulted in a darker surface of the product. Moreover, as the temperature increased, the process of protein denaturation and aggregation intensified, which resulted in an increase in the L* parameter [24,30]. Sun et al. [8] found that as the temperature and heating time of beefsteaks increased, so did the values of the L* and b* parameters, whereas the value of the a* parameter decreased.

In our study, there were no statistically significant differences between the time variants (6, 8, 10, 12, 14, 16, 18 h) in the colour parameters a*, b*, and C*, but the autoclave-cooked samples had higher values of the a*, b*, and C* parameters than the other samples (Table 2). The meat heating times of 10 h and 14 h at 60 °C had no significant effect on the hue angle (H*). On the other hand, the lowest value of the H* was found for the raw meat and meat cooked in an autoclave. These results were different from those obtained by other researchers because the proportion of the red colour (a*) in the cooked meat was inversely proportional to the degree of myoglobin denaturation. The myoglobin denaturation process starts at 60 °C. Sanchez del Pulgar et al. [28] examined the effect of heating temperature on the meat colour and found that the cooked meat at higher temperatures had a lower proportion of the red colour at 80 °C than at 60 °C for various time variants. According to Sanchez del Pulgar et al. [28], the colour saturation depends on the concentration of myoglobin, the level of degradation, and the degree of protein denaturation in meat. Vaudagna et al. [31] found a similar relationship between the colour parameter a* and SV-cooked beef heated at 50–65 °C for 90–360 min.

In our study, there were lower values of the L* parameter but higher values of the a*, b*, and C* parameters in the pork cooked in the autoclave. The changes in these parameters may have been caused by the higher pressure inside the vacuum bag (about 0.2 MPa) and higher temperature (121.1 °C).

The total colour difference (ΔE) value was used to describe the change in the colour values of meat samples, in which the higher the ΔE value, the greater the difference between two measured samples (Table 2). Heat treatment changes the colour of the meat. Therefore, as a control sample, meat cooked for 6 h at 60 °C was selected and the colour of the remaining samples was compared with it. The calculated results allow us to conclude that the time spent heating the samples in the same thermal conditions does not significantly cause further change in the colour of the meat. Heating the meat in the autoclave at 121.1 °C caused a significant change in the colour of the muscle (ΔE = 12.60) (Table 2).

As mentioned before, changes in meat redness (a*) after cooking mainly result from the content of myoglobin and the degree of its denaturation. However, the colour of the muscle tissue is determined not only by the amount of myoglobin, but also by the direction and scope of its chemical transformations. During thermal processing myoglobin and its derivatives are transformed into myochromogens. Myochromogen, which is a denatured form of myoglobin, is red. Heating muscles at a high temperature under elevated pressure increased the degree of myoglobin denaturation and reduced its solubility. As a result, there was less myoglobin in thermal leakage. Despite the high temperature but short heating time (0.5 h), the myoglobin may not have been completely denatured. The lower water content resulting from the increased weight loss (Table 1) caused an increase in the dry matter content and, consequently, a higher concentration of pigments on the muscle surface than in the SV-cooked meat.

The higher values of the b* parameter in the cooked meat can be attributed to an elevated level of metmyoglobin (the oxidised form of myoglobin), which results in a more brownish colour [32]. Obviously, oxidation had a limited effect on the colour due to the use of vacuum bags. The differences in the intensity of this process in the SV-cooked meat and the AC-cooked meat were caused by pressure and temperature. The degree of oxidation is also influenced by pH. Higher temperature and higher pH accelerate the oxidation process. Our research (the results have not been presented in this manuscript) showed that the cooked meat under increased pressure and temperature had a higher pH than both the raw meat and the meat cooked SV at 60 °C for 4 h (AC, 6.07; raw, 5.78; SV, 5.89). Generally, an increase in pH was caused by greater protein denaturation.

Brewer et al. [33] observed that the higher pH of the meat treated under high pressure was correlated with a lower L* value, which resulted in a darker colour of the meat. The results obtained by these researchers may explain why our instrumental colour analysis showed a lower L* value in the AC-cooked muscles than in the SV-cooked muscles.

Generally, there are hardly any studies investigating the effect of cooking meat under elevated pressure and at a high temperature on its physical properties. There have been various publications on the properties of meat subjected to non-thermal high hydrostatic pressure (HHP) treatment followed by heat treatment. However, there are differences in the molecular mechanisms responsible for changes in the properties of cooked meat that has been subjected to the HPP treatment and the properties of pressure-cooked meat. The explanation of our results is hypothetical and requires further research.

### 2.4. Thiamine

Apart from the taste, pork loin is a good source of various nutrients affecting the functions of the human body. It is not only rich in protein and micronutrients such as easily absorbable iron, selenium, zinc, and bioactive compounds (coenzyme Q10 and creatine), but it is also the best source of B vitamins (B1, B2, B6, and B12) among all types of meat. The content of vitamin B1 in pork is 4–5 times greater than in other types of meat [34].

As cooking is an integral part of the processing and preparation of meat products, it significantly affects not only the quality and sensory characteristics, such as the texture and taste of finished products [20,21], but it may also cause undesirable changes, reducing the nutritional value and bioavailability of various components, including vitamins. The loss of vitamins and minerals in cooked meat is caused by molecular interactions, which take place during heat treatment.

The temperature and duration of heat treatment are the main factors affecting the loss of vitamins [18,35]. Therefore, some cooking methods, such as prolonged SV cooking, may cause a large loss of vitamins, whereas shorter cooking time may reduce this loss [36] as the results from the analysis of the data in our experiment show (Table 3).

As can be seen, as the pork SV heating time increased, the retention of vitamin B1 in the muscle decreased. The highest content of thiamine was found in the sample cooked for 6 h, whereas the lowest was found in the one cooked for 18 h.

The fact that the content of vitamin B1 in the thermal leakage was increasing for up to 12 h and remained at the same level for up to 14 h shows that thiamine was released from the muscle. The decrease in the content of thiamine within the time range of 14–18 h was most likely caused by its thermal degradation as a result of longer heating time [35,36].

The effect of high temperature on the vitamin B1 content was clearly visible in muscles cooked under pressure and at high temperature. The content of thiamine in the meat cooked in the autoclave was relatively lower than its content in the meat cooked SV for up to 14 h.

As a consequence of the loss of vitamin B1 in the muscles as a result of the thermal leakage of liquids, its content in dry matter also decreased throughout the heating time range. The highest content of thiamine in the dry non-fat matter was found in the meat cooked SV for 6 h. The content of vitamin B1 in the muscle cooked in the autoclave was comparable to its content in the samples cooked for 14 h.

The loss of vitamins during cooking cannot be avoided. However, some thermal processing techniques significantly influence the content of B vitamins, including thiamine (B1) [37,38]. As results from reference publications show, in comparison with traditional cooking the SV method enables a greater retention of B vitamins, including thiamine (B1) [23,39], due to lower losses. However, the greatest loss of vitamin B1 takes place during the sterilisation of canned food. During traditional cooking and stewing, the loss is about 50–70% [40]. Some of the vitamin contained in the product diffuses into the thermal leakage. When fat is used for frying, the loss of thiamine is greater due to the formation of fat oxidation products. This effect can be eliminated during grilling [41].

### 2.5. Rheology

In our study, the effect of the time of SV heating of pork at 60 °C on its rheomechanical properties was analysed. Dynamical mechanical analysis (DMA) was applied to determine rheological properties. In this method no samples are destroyed, and measurements are not repetitive. It seems to be one of the most reliable sources of information on the properties and quality of meat at different stages of technological processing leading to the end product. In particular, the rheomechanical properties of meat products at different technological stages indicate both their physicochemical state and their structure [42].

Meat is muscle tissue made from muscle cells, which comprise muscle fibres made up of myofibrils and connective tissue. These two structural components of the muscle mainly affect its rheological and textural properties [43]. The colloidal solution of sarcoplasmic proteins, which is muscle juice filling the interior of the fibres and the spaces between the myofibrils, is an equally important structural element affecting the rheological and textural properties of meat. Physically, muscles can be treated as a dispersion system composed of two phases, i.e., a hydrocolloid continuous phase (an aqueous colloidal solution of proteins and real low-molecular-soluble compounds) and a dispersed phase, which is composed of muscle fibres containing insoluble myofibrillar proteins and connective tissue proteins, mainly collagen.

**Figure 1:**
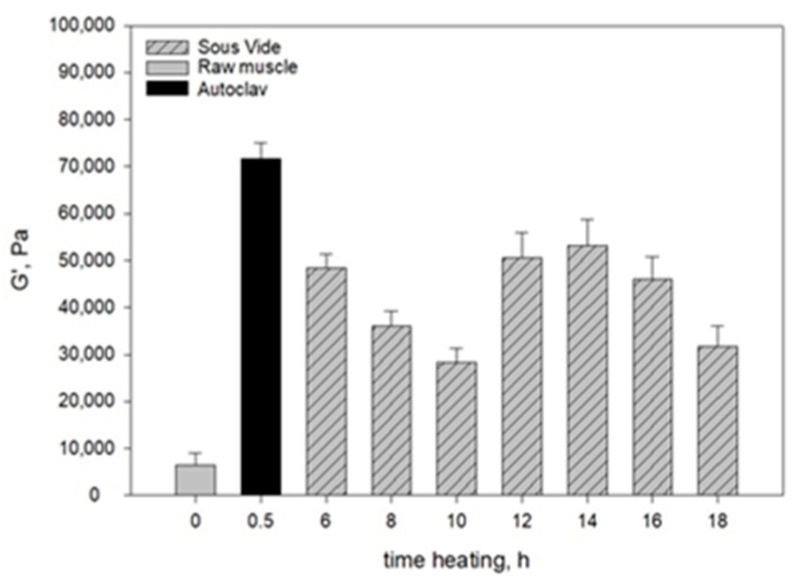
The modulus of elasticity (*G*’) of the systems. The values are expressed as means ± SD (*n* = 6, *p* < 0.05).

**Figure 2:**
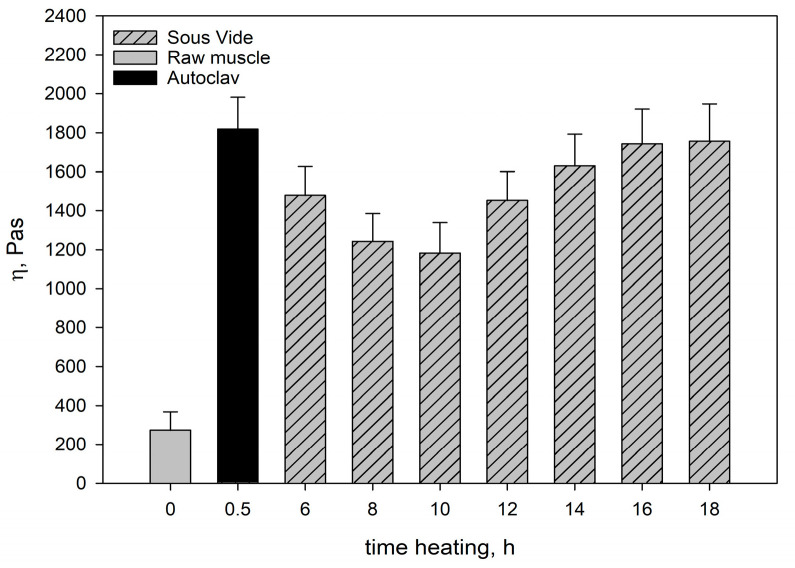
The dynamic viscosity (*η*) of the systems. The values are expressed as means ± SD (*n* = 6, *p* < 0.05).

Figure 1 and Figure 2 show the moduli of elasticity (*G*’) and dynamic viscosity (*η*) of: raw pork, SV-cooked pork, and pork cooked in the autoclave. The moduli of elasticity and dynamic viscosity of the SV-cooked meat were determined as a function of heating time.

**Figure 3:**
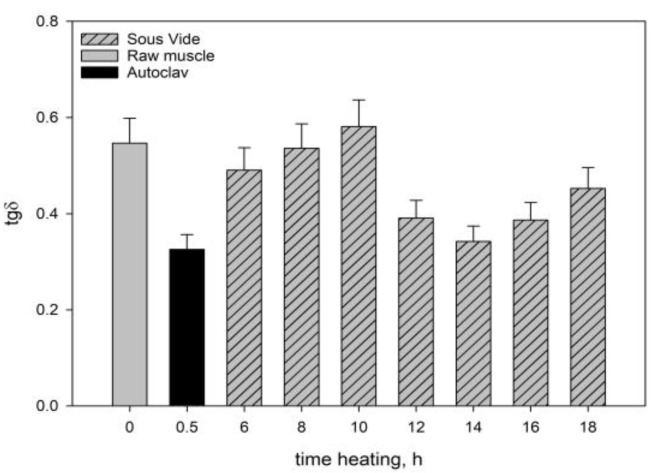
The loss tangent (*tgδ*) of the systems. The values are expressed as means ± SD (*n* = 6, *p* < 0.05).

The rheological tests showed that the raw muscle (*Longissimus dorsi* muscle) had a lower *G*’ value than the heated muscles (Figure 1), because a viscous colloidal solution of sarcoplasmic proteins filling the spaces inside and between the cells in the raw muscle dissipated mechanical energy and limited its spread. The connective tissue and myofibrils containing undenatured myofibrillar proteins (myosin and actin) and collagen contained in the connective tissue were characterised by low stiffness. This resulted not only in lower *G*’ values (Figure 1), but also in a lower value of dynamic viscosity (*η*) (Figure 2). In consequence, there was an increase in the ability to dissipate mechanical energy *tgδ* (Figure 3).

The rheological properties of dispersion systems are determined by the rheological characteristics of the continuous phase, the deformability of the dispersed phase, and the interactions between these phases [44]. Therefore, during the heating of the muscles, the molecular changes occurring within the structural units of the muscle tissue, both in the myofibrillar and connective tissue, are extremely important for the rheological properties [16,25,45].

The changes occurring during muscle heating result in structuring within the hydrocolloid continuous phase filling the spaces inside and between cells, the aggregation and cross-linking of myofibrillar proteins extracted into hydrocolloid, and the gelation of partially dissolved collagen. The denaturation of myofibrillar proteins in muscle fibres causes their stiffening. In consequence, after heating muscles have a more compact structure than raw muscles, whereas all dispersion components (hydrocolloid and muscle tissue) are more strongly bound to each other. This is manifested not only in the increase in the value of rheomechanical parameters (*G*’ and *η*) (Figure 1 and Figure 2), but also in the values of texture determinants (Table 4).

Spatial structures formed by the elastic associations of myosin and actomyosin have a greater capacity for elastic reaction under mechanical influence than the hydrogel resulting from the thermal transformations of the, mainly sarcoplasmic, soluble proteins [46].

The rheological and textural properties of heated muscles are shaped by the processes of protein denaturation and the resulting spatial structures, with the density of the network segments being temperature-dependent.

The approximate relationship between the modulus of stiffness of the highly elastic spatial networks of polymers and the concentration of segments in these networks is determined by the following relation [47]:

*G*′ ≅ *n_s_RT* (1)

where:

*n_s_*: concentration of segments;
*R*: gas constant;
*T*: temperature.

This relationship implies that at a given protein concentration, the increase in stiffness of the system (hydrocolloid) at a given temperature occurs as a result of the expansion of the spatial network nodes. New molecule segments are bound as a result of the interaction between protein polypeptide chains.

After heating the muscle systems for 6–10 h, there was a noticeable decrease in their elastic properties (*G*’) and a corresponding increase in the ability to dissipate mechanical energy (*tgδ*) (Figure 1 and Figure 3) in consequence of the change in the saturation of the hydrocolloid with sarcoplasmic and myofibrillar proteins. As a result, the cross-linking of spatial protein matrices decreased.

The change in the protein saturation of hydrocolloids was caused by structural changes after the heating of, mainly, the muscle fibres (the shrinkage and swelling of myofibrils) and, partly, the connective tissue [22]. As a result, as the heating time increased, the water-holding capacity of the muscles decreased, whereas the exudation of the solution containing mainly sarcoplasmic proteins and partially solubilised collagen increased (Table 1).

As results from the analysis of the data obtained in our study show, the heating time at a constant temperature was one of the main factors responsible for the rheological properties of cooked muscles. It significantly influenced the course and intensity of the molecular processes occurring within the structural units of the muscle tissue, which affected these properties. Generally, at up to 10 h of heating, myofibrils contract transversely and swell, whereas sarcoplasmic proteins gelate [48]. Collagenase, an enzyme contained in sarcoplasmic proteins, causes partial solubilisation of collagen [43] and a major part of the connective tissue. Proteolytic enzymes also contribute to changes in the structure of the connective tissue. After 10 h there are further transformations in the connective tissue structures and muscle fibres. The connective tissue network and muscle fibres contract longitudinally. As a result, proteins in myofibrils have a more aggregated, dense structure, whereas the elasticity of muscle fibres increases. This is reflected by the values of rheological determinants. Within the range of the meat heating time analysed in our study, the moduli of elasticity (*G*’) (Figure 1) were greater than in the meat cooked for 10 h. The ability to dissipate mechanical energy (*tgδ*) decreased noticeably (Figure 3). At the same time, the degree of cross-linking of the hydrocolloid phase increased. This effect was caused by the progressing gelatinisation of solubilised collagen as the heating time increased from 12 h to 16 h and by the increase in the content of myofibrillar proteins extracted into the hydrogel [46,49]. These proteins not only compensate for the loss of sarcoplasmic proteins and dissolved collagen due to thermal leakage, but also improve the elastic properties of the hydrocolloid. This was manifested in an increase in the value of dynamic viscosity *η* (Figure 2) of the muscle systems, observed within that time interval. The slight decrease in the elastic properties of the meat cooked for 18 h may have been caused by the expulsion of some non-aggregated proteins contained in the hydrocolloid into the extracellular spaces as a result of the pressure exerted by the shrinking connective tissue. Thus, these proteins did not participate in the formation of the protein matrix, nor did they have any effect on its elastic properties.

The rheomechanical properties of the meat cooked under elevated pressure (0.2 MPa) and at a high temperature (121.1 °C) were compared with those of the SV-cooked meat. The values of the modulus of elasticity (*G*’) and dynamic viscosity (*η*) in the meat cooked in the autoclave were significantly greater than in the SV-cooked meat, regardless of the heating time (Figure 1 and Figure 2). The high values of the modulus of elasticity (*G*’) but significantly lower energy dissipation capacity (Figure 3) show that the elastic properties of the meat cooked in the autoclave were much greater than those of the SV-cooked meat. 

In the muscles cooked at a high temperature (121.1 °C), the changes in the myofibrils and in the intramuscular connective tissue were greater than in the SV-cooked muscles at 60 °C. These changes were caused by the denaturing processes/transitions of myofibrillar and connective tissue proteins, mainly collagen. As a result of their gelation, the cross-linking density of the intermuscular hydrocolloid continuous phase increased significantly. Due to the greater aggregation of proteins in the muscle fibres, they were stiffer than the SV-cooked muscles at 60 °C. This effect was manifested in higher values of the rheomechanical parameters (*G*’, and *η*) (Figure 1 and Figure 2) and a lower energy dissipation capacity (Figure 3).

The marked increase in the *G*’ value after 30 min of isothermal heating at 121.1 °C resulted from the denaturation of actin, the second main myofibrillar protein, which probably forms a gel network between denatured actin and myosin, and with other actin molecules, which additionally increases the elasticity of the spatial network of proteins.

Due to the fact that actin begins to denature at 74 °C [49,50], these proteins were in their native state in the SV-cooked muscles at 60 °C and did not have any influence on their elastic properties.

### 2.6. Texture Analysis

#### 2.6.1. WBSF

The shear force test (Warner–Bratzler shear force: WBSF) and texture profile analysis are classic instrumental methods of meat tenderness (hardness) assessment.

The shear force test measures the maximum force (N) as a function of knife movement (mm) and compression to shear (cut off) a meat sample (MPa). The result of this measurement indicates the hardness (toughness) of meat.

The maximum force observed during the shear test was selected to characterise the texture of the samples (Table 4).

In the shear force and shear work tests, all of the samples were characterised by similar dependencies in relation to the time variants under analysis. Among the SV-cooked samples, the pork cooked for 6 h was characterised by the greatest shear force and shear work values, which were significantly different from the values observed in the other heated samples.

As the heating time increased up to 12 h, there was a statistically significant decrease in the values of both parameters (shear force and shear work). During heating for 14 h there was a noticeable increase in the shear force and shear work. When the heating time was extended to 18 h, the values of both parameters decreased significantly (Table 3).

The shear force values noted in our tests were similar to the results of the experiment conducted by Christensen et al. [51], in which the shear force values of the pork Longissimus dorsi muscle ranged from 12.6 to 41.1 N and decreased as the cooking temperature increased. The influence of the cooking time was not uniform and depended on the cooking temperature. However, in an experiment conducted by Vaudagna et al. [31], the mean shear force values decreased as the cooking temperature increased, but the meat cooking time did not have significant effect on the mean shear force values. The observations made by Vaudagna et al. in an experiment on the influence of beef heating time on shear force values were different from the observations made in our study. This may have been caused by the fact that our pork heating times were much longer than the beef heating times used by Vaudagna and his team.

Bertola et al. [52] noted the lowest shear force values (WBSF) during the heating of small pieces of beef (diameter: 1.5 cm; length: 2 cm) at 60 °C and 64 °C. Low values of shear force are desirable in cooked meat because they reflect a greater tenderness of the ready-to-eat product. Meat tenderness is influenced by myofibrils and connective tissue proteins and their transformations during heat treatment, mainly the denaturation of myofibrillar proteins (myosin and actomyosin) and the solubilisation of collagen [23].

Hwang et al. [14] observed that the shear force of pork tenderloin was affected by the process temperature, whereas the process time had a significant effect on the meat tenderness only at 50 °C. The researchers suggested that the tenderness of meat cooked for a longer period of time (24 h vs. 12 h) was enhanced by the activity of intrinsic proteases, which remained active at such a low temperature. The thermal inactivation of proteases at temperatures higher than 55 °C may have been the reason why the heat-induced structural changes in meat proteins had a significant influence on meat tenderness.

Similarly, Jeong et al. [24] observed that the cooking temperature had a significant effect on the shear force of pork ham cooked SV at 61 °C and 71 °C, whereas the effect of the cooking time was observed at 71 °C. Increased meat tenderness during cooking is caused by the conversion of collagen to gelatine [49]. Ismail et al. [53] observed increasing collagen solubility in beef cooked at 45 °C, 65 °C, and 75 °C. Vasanthi et al. [54] observed that collagen solubility tended to increase along with cooking temperature (80–100 °C) and time (30–60 min). The increase in the degree of collagen dissolution and the degree of denaturation of muscle proteins are important factors affecting the textural properties of muscles, as evidenced by the analysis of the texture parameters of the muscles heated in the autoclave.

In the muscles cooked in the autoclave at 121.1 °C, collagen was dissolved and myofibrillar proteins were denatured to a much greater extent than in the SV-cooked muscles heated at 60 °C. As a result, the myofibrillar fibres were stiffer. Gelled collagen increases the cross-linking density of the intermuscular hydrocolloidal phase. As a result, the muscles heated in the autoclave had a more compact structure than the SV-cooked muscles, whereas all dispersion components (hydrocolloid and muscle tissue) were more strongly bound to each other, as evidenced by an increase in the shear force and shear work (Table 4).

#### 2.6.2. Texture Profile Analysis (TPA)

TPA provides more information on the texture of meat products than shear force, which is a useful indicator of initial meat tenderness [32]. The texture profile attributes are listed in Table 4.

During the SV heating of pork, hardness I and II decreased gradually as the cooking time increased from 6 h to 10 h. The decrease in both parameters was statistically significant. Then, their values increased until 14 h of heating time was reached. After 14 h of heating, hardness I and II tended to deteriorate again until 18 h of heating time had passed (Table 4). Changes in meat tenderness and hardness during SV cooking are caused by heat-induced changes in the connective tissue and muscle fibre proteins. When the heat contained inside the vacuum bag comes into contact with the moist content of the bag, the connective tissue begins to dissolve. It results in partial solubilisation and gelation of collagen, which increases meat tenderness. The denaturation of myofibrillar proteins causes the stiffening of muscle fibres, which increases meat hardness [49].

As was the case with the shear force and work, during the heating of pork for 14 h there was an increase in hardness I and II (Table 4). This may have been caused by the heterogeneous structure of the muscle tissue, the amount of connective tissue (collagen), tendons, and intramuscular fat in the muscle under analysis. Roldan et al. [29] observed that when lamb was heated for a longer period of time at the same temperature, the hardness of the sample decreased. The meat was cooked at 60 °C, 70 °C, and 80 °C for 6 h, 12 h, and 24 h.

Polak et al. [55] studied the effect of SV technology on beef quality. The researchers observed that meat samples cooked for 30 h at 64 °C, 68 °C, and 72 °C were less hard than beef samples cooked for 24 h. They concluded that a longer SV heating time contributed to the softening of muscle, which may have been caused by the complete dissolution of collagen [49].

The highest chewiness was observed in the SV-cooked pork after 6 h of heating. As the cooking time increased from 6 to 10 h, the chewiness of the pork decreased. It remained at a similar level up to 18 h of heating and there were no statistically significant differences. This may have been caused by the fact that many enzymes are denatured during SV cooking at 55–60 °C, but some collagenases are active and can significantly increase meat tenderness after more than 6 h of heating [56]. Roldán et al. [29] observed a similar effect of the heating time on chewiness. They found that cooking time reduced chewiness. However, when meat was heated at 60 °C for 6, 12, and 24 h, chewiness decreased only after the heating time was extended to 24 h.

In our study, the meat cooked in the autoclave had the highest elasticity, and its values differed statistically significantly from the other variants of the experiment. The meat samples heated for 12, 14, and 18 h had lower elasticity than the meat cooked for 8 and 10 h, and they did not differ statistically significantly from each other. The highest elasticity was observed in the SV-cooked pork heated for 6 h. The value of this parameter did not differ statistically significantly from that of the meat cooked for 10 h. There were no statistically significant differences in elasticity between the meat samples heated for 12, 14, 16, and 18 h.

Elasticity is defined as the ability of a deformed product to quickly return to its natural shape [42]. Sánchez del Pulgar et al. [28] observed that the heating of pork at 60 °C for 5 or 12 h did not cause any significant changes in hardness, elasticity, cohesiveness, or chewiness. The values of these parameters decreased only in the samples heated for 12 h at 80 °C. Jeong et al. [24] found a slight difference in the elasticity and consistency of SV-cooked meat samples as the temperature increased from 61 °C to 71 °C. They also observed that when the heating time was extended from 45 to 90 min, there were slight changes in springiness and elasticity. Similarly, although in our study the heating times were much longer, the changes in elasticity were minimal.

In comparison with the SV-cooked muscles, those cooked in the autoclave (121.1 °C) had higher values of textural parameters determined both with the WBFS and TPA methods within the entire range of durations.

The molecular mechanisms responsible for rheological (described above) and textural changes occurring in muscles during isothermal heating at specific temperatures are similar. At a high temperature (121.1 °C) and elevated pressure, changes in myofibrils, connective tissue proteins, and their transformations during heat treatment occur with much greater intensity than at the temperature used for SV cooking (60 °C). In consequence of heating the muscles at different temperatures, they differ in the degree of bonding of the dispersion components (hydrocolloid and muscle tissue). This is manifested not only in differences in the values of texture determinants (Table 4), but also in the values of rheomechanical and parameters (*G*’ and *η*) (Figure 1 and Figure 2).

#### 2.6.3. Sensory Evaluation

Scientific sensory evaluation by qualified panellists is a simple and reliable method providing useful results for consumers. For an average consumer, this is the only way to decide whether they should buy or eat a particular product. The results of the sensory evaluation are listed in Table 5.

Consumers prefer tender and juicy meat. Texture and juiciness are important meat quality criteria. Meat should be cooked for long enough to be sufficiently tender. The sensory analysis (Table 5) showed that as the cooking time increased from 6 h to 12 h, the juiciness and tenderness of the meat increased. The values of these parameters remained at the same level for the meat samples cooked for 16 and 18 h, except for the samples cooked for 14 h, which received lower scores (Table 5).

The meat sample cooked for 12 h had the highest sensory scores in all parameters (juiciness, tenderness, smell, and flavour). The meat sample cooked in the autoclave had the lowest sensory scores.

These results show that a longer SV cooking time than 12 h may negatively affect the quality and sensory characteristics of meat, but it may still be acceptable to consumers.

## 3. Materials and Methods

### 3.1. Materials 

Three pork loins (*Longissimus lumborum* muscles) were purchased for our study. The research material came from PIC commercial hybrids (5–6-month-old female pigs weighing about 110 kg). It was collected from a local meat supplier 24 h after the animals had been slaughtered. Next, it was transported to a laboratory under refrigeration, vacuum packed, and stored at 4 °C for 2 days. After the storage, each muscle was trimmed and sliced into pieces of a similar size (8 × 8 × 2.5 cm slices) to avoid differences in cooking. The slices were individually weighed and vacuum packed in PA/PE bags (15 mm polyamide/60 mm polyethylene; heat resistance of −20 °C/+110 °C; Hendi, Lamprechtshausen, Austria) with a chamber vacuum sealer (STALGAST 691310, Radom, Poland). An SV cooking device (Hendi Sous-Vide Machine GN1/1, Poznań, Poland) was used in the experiment. The device consisted of a 20 L water tank with a thermostat and a circulation system, which enabled water to be heated up to 90 °C. The meat was SV cooked at a constant temperature of 60 °C in vacuum bags for 6, 8, 10, 12, 16, and 18 h and at 121.1 °C in the A-125 E autoclave (Jugema, Środa Wielkopolska, Poland) for 30 min. Three slices were randomly selected (one slice from each of the purchased muscles) for each variant of the experiment: the raw muscle and the samples heated for 6, 8, 10, 12, 16, and 18 h, and at 121.1 °C in the autoclave. After the cooking time, the samples were removed from the hot water and they were immediately dipped in water with ice (for 0.5 h) to cool down at 4 °C. Then they were stored at 4 ± 1 °C until analysis. The entire experiment was conducted in two independent replicates, with meat purchased (twice) from the same supplier. The samples for the replicated experiment were prepared in the same manner.

### 3.2. Methods

#### 3.2.1. Primary Composition Analysis

##### Protein Content

The protein content was measured with the Kjeldahl method [57] by determining the content of nitrogen in the sample. A total of 0.5 g of the sample was weighed on an analytical balance and placed in a test tube. Next, two Kjeltabs tablets or 0.8 g of CuSO_4_, 7 g of K_2_SO_4_, and 12 cm^3^ of concentrated H_2_SO_4_ were added as a catalyst. The mixture was burnt in a Digeston oven at 425 °C for 45–60 min. The resulting solution was then distilled in a Kjeltec apparatus with 33% NaOH. The distillate was placed in a flask with 20 cm^3^ of 4% boric acid and a Tashiro indicator, and titrated with 0.1 nH_2_SO_4_ solution.

The formula below was used to calculate the protein content:

$$X=\frac{1.4\times a\times 6.25}{10\times b}$$

where:

*X*: the protein content (%);
*a*: the amount of 0.1 nH_2_SO_4_ (cm^3^) consumed for titration;
*b*: the weighed amount (g);
1.4: the amount of nitrogen corresponding to 1 cm^3^ of 0.l nH_2_SO_4_;
6.25: the conversion factor for the amount of total nitrogen to protein.

##### Total Water Content

In order to measure the water content, a 5 g sample (weighed with an accuracy of 0.001 g) was dried in an oven at 105 °C for 6 h. Then, it was placed in a desiccator until it reached room temperature. Next, it was weighed and placed back in the dryer for 1 h. This procedure was repeated until the difference in weight between the individual measurements was equal to 0.00l g [58].

The formula below was used to calculate the water content:

$$X=\frac{\left(a-b\right)\times 100}{c}$$

where:

*X*: the water content (%);
*a*: the mass of the weighed amount with blotting paper before drying (g);
*b*: the mass of the weighed amount of the blotting paper after drying (g);
*c*: the mass of the weighed amount (g).

##### Cooking Loss

Cooking loss was calculated from the difference in the meat weight before and after heat treatment. Each sample was dried before weighing. The weighing accuracy was 0.01 g. The formula below was used to calculate the cooking loss:

$$CL(\%)=\frac{\left(m_{R}-m_{h}\right)\times 100}{m_{h}}$$

where:

*CL*: the cooking loss (%);
*m_R_*: the mass of raw meat (g);
*m_h_*: the mass of cooked meat (g).

##### PH Measurement

Ten grams of comminuted raw meat samples was homogenised with deionised water (1:10) in an HO_4_/A homogeniser (Edmund Bühler GmbH, Hechingen, Germany) for 2 min at 5000 rpm. After equilibrium was reached, the measurements were recorded with a portable digital HI 99,161 m (Hanna Instruments, Eibar, Spain) equipped with an FC2023 glass electrode. The electrode was calibrated with pH 7.0 and 4.0 buffers (Merc, Darmstadt, Germany).

##### Fat Content

The fat content was measured with the Soxhlet method [59]. A sample of about 5 g was wrapped in blotting paper to form a thimble, which was placed in a dryer. After drying to a constant mass, the sample was placed in a Soxhlet apparatus. Fat was extracted with anhydrous petroleum ether. After the extraction, the samples were weighed on an analytical balance and the fat content was calculated according to the formula below:

$$X=\frac{\left(a-b\right)\times 100}{c}$$

where:

*X*: the fat content (%);
*a*: the weight of the thimble before extraction (g);
*b*: the weight of the thimble after extraction (g);
*c*: the mass of the weighed amount (g).

##### Total Thiamine Content

The content of thiamine was measured with the thiochrome method by analysing the quantitative changes of the free form [60]. The method is based on the oxidation of thiamine to thiochrome and a fluorimetric measurement of its amount at an excitation wavelength of *λ* = 365 nm, and the secondary filter with a maximum permeability at a wavelength of *λ* = 435 nm. First, the sample underwent acid and enzymatic hydrolysis. Then, the obtained thiamine was released from the substances interfering with its oxidation to thiochrome. Next, it was measured fluorimetrically. The sample was ground in a mortar to obtain a uniform mass. About 5 g was weighed and transferred to a 500 mL conical flask with a ground glass stopper. Then 200 mL of 0.1 N HCl was poured out. The closed flask with the product and 0.1 N HCl was heated in a boiling water bath for 0.5 h. A 10% diastase solution (Merck, Germany) was added for enzymatic hydrolysis. Next, the samples were shaken at 45–50 °C for 2 h. Then, they were filtered into a 250 mL flat-bottomed flask. The hydrolyte was diluted with distilled water up to 250 mL. A total of 25 mL of the hydrolyte was collected and placed on a column filled with Amberlit IRC-50 resin (Sigma, Sigma-Aldrich, Darmstadt, Germany). At the next stage of the analytical procedure, thiamine was converted to thiochrome with 5 mL of isobutanol and 1.2 mL of an alkaline solution of potassium ferrocyanide. Fluorescence was measured with a Jenway Model 6200 fluorimeter (Dedham, UK) at a wavelength of λ = 436 nm. After measuring the fluorescence in the sample, thiochrome fluorescence was quenched by adding 1 drop of 18% HCl to the cuvette with the sample.

The thiamine content was read from the standard curve and converted to the weight of 100 g:

$$T_{o}=\frac{c\times 125\times 100}{M}$$

where:

*T_o_*: the total thiamine (μg/100 g);
*c*: the concentration of total thiamine (μg/2 mL) read from the standard curve;
*M*: the mass of the test sample (g).

#### 3.2.2. Instrumental Colour Assessment

The physical colour parameters, L*, a*, and b*, of the cooked meat samples were assessed instrumentally in the CIE system, where:

L*: lightness (0: a perfectly black body; 100: a perfectly white body);
a*: redness (when positive), green (when negative), or grey (when 0);
b*: yellowness (when positive), blue (when negative), or grey (when 0).

The colour components were measured with the reflection method within the range of visible light (400–700 nm). The Spectro-Pen LMG 161 spectrophotometric apparatus (Dr Lange, Munich, Germany) was used for the measurements. Standard observer’s settings were applied, i.e., angle of incidence 10°, light D65. The apparatus was calibrated each time before measurements, allowing for the scattered light and white reference LZM 224 Standard 3125 of *X*cal = 92.1, *Y*cal = 97.2, *Z*cal = 103.9.

The values of hue (H* = tan^−1^(b*/a*)) and chroma (C* = (a*^2^ + b*^2^)^0.5^) were calculated. Additionally, the total colour difference (ΔE) between the control (sample 6 h) and test samples was calculated according to formula,

Δ*E* = [(L*_0_ − L*_1_)^2^ + (a*_0_ − a*_1_)^2^ + (b*_0_ − b*_1_)^2^]^0.5^

where:

Δ*E*: total colour difference;
L*_0_, a*_0_, b*_0_: means of colour parameters determined for the control samples;
L*_1_, a*_1_, b*_1_: means of colour parameters determined for the test samples.

In the interpretation of the results, it was assumed that:

when 0 < Δ*E* < 1, the observer does not notice the difference;
when 1 < Δ*E* < 2, only an experienced observer may notice the difference;
when 2 < Δ*E* < 3.5, an inexperienced observer notices the difference;
when 3.5 < Δ*E* < 5, a clear difference in colour is noticed; and
when 5 < Δ*E*, an observer notices two different colours.

#### 3.2.3. Rheological Properties 

The rheological properties were measured with a dynamic mechanical thermal analyser (DMWT) (COBRABiD—Poznań, Poland). A parallel plate with a 50 mm diameter and 2 mm gap measuring system was used. The following components of the complex modulus of elasticity were calculated: modulus of elasticity (*G*’), loss tangent (*tgδ*), and dynamic viscosity (*η*). The *G*’ is associated with the part of potential deformation energy maintained in the course of periodical deformations. The *tgδ* is a measure of internal friction. It describes the relative quantity of energy dissipated in the material in the course of one deformation cycle. The frequency of vibrations in the systems amounted to 1.2 Hz. The rheological properties of the systems were tested at 22 °C. The temperature of the chamber and measurement plate was measured with an accuracy of ±0.2 °C. The linear viscoelastic region of each sample was taken into account. The rheological properties of the samples were measured in six replicates.

#### 3.2.4. Texture Analysis

##### Warner–Bratzler Shear Blade Test (WBFS)

The shear force was measured with a Warner–Bratzler blade. The samples used in the tests were 1 × 10^−2^ m in diameter and 1.5 × 10^−2^ m in height. The head travel speed was 1.5 m/min. The maximum shear force (firmness) (N) and shear work (toughness), as the area under the curve (Ns), were read from the recorded shear forces. The parameters were measured in 10 replicates at room temperature.

##### Texture Profile Analysis (TPA)

The double compression method was applied for texture profile analysis (TPA) of the pork samples after different durations of SV heating [44]. Samples of 1 × 10^−2^ m in diameter and 1 × 10^−2^ m in height were subjected to double compression to 50% of their original height with a TA.XT.plus texturometer (Hamilton, MA, USA) equipped with a P/10 attachment and HDP/90 object table. The head used in the TPA moved at a speed of 1.5 × 10^−2^ m/min. The following texture parameters, according to Bourne [44], were determined from a general force–strain relationship plot during two compression tests: hardness I and II (N), springiness (mm), elasticity (mm), gumminess (N), and chewiness (Nmm). The parameters were measured in 10 replicates at room temperature.

##### Sensory Evaluation

The sensory evaluation of the cooked pork sample was conducted by a trained and experienced team of eleven experts with established and proven sensory sensitivity (in accordance with the following standards: PN EN ISO 8586) [61]. The samples were evaluated for juiciness, tenderness, smell, and flavour according to a five-point scale (1: the lowest score; 5: the highest score). Intensity ranged from 1 point (very slight) to 5 points (very strong). Desirability was ranked from 1 point (undesirable) up to 5 points (very desirable) with an accuracy of 0.5 points.

#### 3.2.5. Statistical Analysis 

The cooking experiments were conducted in 6 replicates for each heating duration. The results of the basic composition (protein, fat, water, fat-free dry matter, cooking loss, thiamine content, pH), texture, rheomechanical, and colour tests were expressed as means ± standard errors. The STATISTICA 10 (Statsoft, Kraków, Poland) software was used for one-way analysis of variance (ANOVA) to investigate the effect of the cooking time on the basic composition, texture, rheomechanical properties, and colour. The significance of the differences was determined with Fisher’s test at a significance level of *p* < 0.05.

## 4. Conclusions

As results from our study show, the duration of SV heating of meat had a significant influence on the course and intensity of molecular transformations occurring in the structural units of the muscle tissue. The intensity of structural changes occurring in the myofibrillar systems and connective tissue, as well as the activity of proteolytic enzymes determined by the heating time, are the main factors affecting the rheological and textural properties of meat. Due to the long heating time, and despite the low temperature of SV cooking, the meat was more tender than the meat cooked briefly at a high temperature in the autoclave. It was also juicier due to the smaller loss of weight during heating, and it retained more nutrients (vitamin B1). After 12 h of cooking the meat at 60 °C, the lowest values of the texture parameters (shear force, elasticity, and chewiness) were recorded.

At the same time, the sensory analysis showed that the meat samples cooked for 12 h had the highest sensory scores for all parameters (juiciness, tenderness, smell, and flavour). The meat sample cooked in the autoclave had the lowest sensory scores. In conclusion, pork loins cooked at low temperatures (60 °C) for a long time are more attractive and acceptable than pork loins cooked briefly at a high temperature.

## Figures and Tables

**Table 1:** The basic composition and cooking loss of pork heated at 60 °C for different times and autoclaved at 121.1 °C.

Variant	Water, %	Fat, %	Fat Free Dry Matter, %	Protein, %	Cooking Loss, %	pH (-)
Raw Pork	73.98 ± 2.80 ^a^	3.42 ± 0.14 ^a^	22.62 ± 1.32 ^a^	22.09 ± 1.35 ^a^	-	5.78 ± 0.01
Pork 6 h	69.39 ± 2.17 ^b^	2.73 ± 0.11 ^b^	27.88 ± 1.45 ^bf^	27.05 ± 1.36 ^bc^	23.19 ± 1.29 ^a^	-
Pork 8 h	67.28 ± 2.10 ^c^	4.95 ± 0.15 ^c^	27.78 ± 1.45 ^b^	27.22 ± 1.37 ^bd^	24.41 ± 1.02 ^a^	-
Pork 10 h	67.66 ± 2.11 ^c^	5.48 ± 0.16 ^d^	26.86 ± 1.44 ^c^	26.70 ± 1.32 ^bc^	24.63 ± 1.11 ^a^	-
Pork 12 h	67.33 ± 1.86 ^c^	5.81 ± 0.18 ^d^	26.95 ± 1.48 ^c^	26.50 ± 1.31 ^c^	25.35 ± 1.04 ^ab^	-
Pork 14 h	67.36 ± 1.82 ^c^	4.01 ± 0.15 ^e^	28.64 ± 1.39 ^d^	28.21 ± 1.45 ^ef^	25.79 ± 1.19 ^ab^	-
Pork 16 h	67.55 ± 2.07 ^c^	3.31 ± 0.14 ^a^	29.16 ± 1.47 ^e^	28.34 ± 1.46 ^e^	26.36 ± 1.18 ^bc^	-
Pork 18 h	67.78 ± 2.12 ^c^	4.27 ± 0.14 ^e^	27.95 ± 1.45 ^f^	27.92 ± 1.38 ^df^	26.72 ± 1.16 ^c^	-
Autoclave	62.80 ± 1.54 ^d^	5.50 ± 0.16 ^d^	31.71 ± 1.51 ^g^	30.85 ± 1.46 ^g^	33.87 ± 1.16 ^d^	-

The mean values ± SD with different letters (a–g) in each column differ significantly (*p* < 0.05).

**Table 2:** The colour of pork samples cooked at 60 °C for various times and the samples cooked in the autoclave at 121.1 °C.

Variant	L*	a*	b*	H*	C*	ΔE
Raw Pork	45.38 ^a^	−2.76 ^a^	6.00 ^a^	0.077 ^a^	6.60 ^a^	453.82
Pork 6 h	75.04 ^b^	0.42 ^b^	10.22 ^b^	10.328 ^b^	10.23 ^b^	Control simple
Pork 8 h	76.04 ^bc^	0.24 ^b^	10.40 ^b^	32.768 ^c^	10.40 ^b^	0.53
Pork 10 h	76.60 ^c^	0.20 ^b^	10.02 ^b^	43.802 ^d^	10.02 ^b^	1.26
Pork 12 h	73.62 ^bc^	0.62 ^b^	11.20 ^b^	5.69 ^e^	11.22 ^b^	1.51
Pork 14 h	74.92 ^bc^	0.20 ^b^	10.00 ^b^	43.627 ^d^	10.00 ^b^	0.06
Pork 16 h	73.66 ^b^	0.16 ^b^	10.22 ^b^	71.204 ^f^	10.22 ^b^	0.99
Pork 18 h	76.28 ^bc^	0.70 ^b^	9.46 ^b^	3.182 ^e^	9.49 ^b^	1.10
Autoclave	68.52 ^d^	4.78 ^c^	20.08 ^c^	0.302 ^a^	20.64 ^c^	12.60

The mean values with different letters (a–f) in each column differ significantly (*p* < 0.05).

**Table 3:** The thiamine content in the muscle and the thermal drip from pork cooked at 60 °C for different periods of time and from pork autoclaved at 121.1 °C.

Variant	Thiamine Content in the Muscle, %	Thiamine Content in the Thermal Drip, %	Thiamine Content in Fat-Free Dry Matter, %
Raw Pork	100.00	-	100.00
Pork 6 h	70.68 ± 0.98 ^a^	3.92 ± 0.22 ^a^	58.00 ± 0.87 ^a^
Pork 8 h	68.02 ± 1.30 ^b^	5.77 ± 0.31 ^b^	56.20 ± 0.98 ^b^
Pork 10 h	61.77 ± 1.34 ^c^	6.80 ± 0.27 ^c^	53.12 ± 1.24 ^c^
Pork 12 h	59.69 ± 1.70 ^d^	7.93 ± 0.26 ^d^	50.13 ± 1.38 ^d^
Pork 14 h	51.72 ± 1.94 ^e^	7.86 ± 0.24 ^d^	40.31 ± 1.45 ^e^
Pork 16 h	46.97 ± 2.26 ^f^	6.91 ± 0.15 ^c^	37.25 ± 1.63 ^f^
Pork 18 h	37.72 ± 2.57 ^g^	4.53 ± 0.12 ^e^	30.11 ± 1.78 ^g^
Autoclave	56.78 ± 2.31 ^h^	7.89 ± 0.21 ^d^	40.08 ± 1.52 ^e^

The mean values ± SD with different letters (a–h) in each column differ significantly (*p* < 0.05).

**Table 4:** The texture parameters in the pork cooked at 60 °C for various times and in the pork cooked in the autoclave at 121.1 °C.

Variant	Shear Force, N	Shear Work, J	Hardness I, N	Hardness II, N	Springiness, mm	Elasticity, mm	Chewiness, Nxmm
Raw Pork	17.82 ^a^	86.76 ^a^	-	-	-	-	-
Pork 6 h	19.79 ^b^	98.57 ^b^	26.55 ^a^	23.47 ^a^	0.46 ^a^	0.46 ^a^	5.81 ^a^
Pork 8 h	17.57 ^a^	85.63 ^a^	19.74 ^b^	17.37 ^b^	0.43 ^ab^	0.41 ^bd^	3.21 ^b^
Pork 10 h	14.98 ^c^	59.28 ^c^	12.92 ^df^	10.29 ^cf^	0.47 ^a^	0.45 ^a^	2.55 ^bc^
Pork 12 h	9.10 ^d^	57.85 ^cd^	14.60 ^de^	12.83 ^cd^	0.41 ^bd^	0.37 ^c^	2.15 ^c^
Pork 14 h	14.75 ^c^	60.29 ^c^	16.32 ^ce^	13.82 ^de^	0.39 ^cd^	0.39 ^bc^	2.22 ^c^
Pork 16 h	10.36 ^d^	55.10 ^d^	14.36 ^e^	12.61 ^cd^	0.37 ^c^	0.38 ^bc^	3.12 ^c^
Pork 18 h	9.99 ^d^	56.55 ^d^	10.85 ^f^	8.79 ^f^	0.40 ^bd^	0.39 ^bc^	1.89 ^c^
Autoclave	19.36 ^b^	108.16 ^e^	18.31 ^bc^	16.62 ^be^	0.61 ^e^	0.41 ^d^	5.15 ^a^

The mean values ± SD with different letters (a–f) in each column differ significantly (*p* < 0.05).

**Table 5:** Results of sensory evaluation of pork heated at 60 °C for various times (points).

Variant	Juiciness	Tenderness	Smell	Flavour
Pork 6 h	1.18 ^a^	1.23 ^a^	3.09 ^abc^	3.18 ^ab^
Pork 8 h	1.77 ^b^	1.86 ^b^	3.18 ^abc^	3.27 ^ac^
Pork 10 h	2.64 ^c^	2.50 ^c^	3.14 ^abc^	3.41 ^ac^
Pork 12 h	4.55 ^d^	4.45 ^d^	3.36 ^a^	3.64 ^c^
Pork 14 h	4.05 ^e^	4.05 ^c^	3.23 ^ac^	3.27 ^ac^
Pork 16 h	4.50 ^d^	4.45 ^d^	3.27 ^a^	3.45 ^ac^
Pork 18 h	4.50 ^d^	4.55 ^d^	2.91 ^bc^	3.50 ^ac^
Autoclave	2.09 ^b^	1.95 ^b^	2.86 ^b^	2.86 ^b^

The mean values with different letters (a–e) in each column differ significantly (*p* < 0.05).

## Data Availability

Not applicable.
